# Diet Quality and Bone Measurements Using HRpQCT and pQCT in Older Community-Dwelling Adults from the Hertfordshire Cohort Study

**DOI:** 10.1007/s00223-018-0445-x

**Published:** 2018-06-21

**Authors:** S. C. Shaw, C. M. Parsons, N. R. Fuggle, M. H. Edwards, S. M. Robinson, E. M. Dennison, C. Cooper, K. A. Ward

**Affiliations:** 1 MRC Lifecourse Epidemiology Unit, University of Southampton, Southampton General Hospital, Southampton, SO16 6YD UK; 2 Portsmouth Hospitals NHS Trust, Portsmouth, UK; 3 Biomedical Research Centre, University of Southampton and University Hospital Southampton NHS FT, Southampton, UK; 4 MRC Elsie Widdowson Laboratory, Cambridge, UK; 5 National Institute for Health Research Musculoskeletal Biomedical Research Unit, University of Oxford, Oxford, OX3 7LE UK

**Keywords:** Diet quality, Older adults, Bone microarchitecture, HRpQCT, pQCT

## Abstract

There are few data describing associations between dietary patterns and bone microarchitecture. This study investigated the relationship between diet quality and HRpQCT and pQCT measures in older adults. Data were available for 184 men and 166 women. Dietary data were collected at baseline (1998–2003) using an administered food frequency questionnaire. A ‘prudent’ diet score (PDS) was identified using principal component analysis and used as an indicator of dietary quality. HRpQCT and pQCT images were acquired at follow-up in 2012, from the non-dominant distal radius and tibia using Scanco XtremeCT and Stratec XCT2000 instrument scanners, respectively. The mean (SD) PDS was − 0.24 (1.23) for men and 0.62 (1.14) for women. In women, a significant positive relationship was found between baseline dietary pattern and total and trabecular area at both the radius and the tibia, measured by HRpQCT. Similar trends were observed with pQCT parameters. Positive associations were observed for tibia total area (38% slice). At the radius, significant positive associations were found for total area (4% slice) and polar strength strain index (33% slice). All relationships remained robust to adjustment. For men, although patterns were similar, there were no significant associations for HRpQCT outcomes. Significant associations were observed for baseline PDS and polar strength strain and total area (66% slice) at the radius, measured by pQCT. Our data suggest that diets high in fruit, vegetables, oily fish and whole grain cereals in early old age are associated with greater bone size but not volumetric bone density or microarchitecture in later life in women.

**Electronic supplementary material:**

The online version of this article (10.1007/s00223-018-0445-x) contains supplementary material, which is available to authorized users.

## Introduction

Diet has long been recognised as an important modifiable factor with a role in the development of bone and its maintenance throughout life. Research investigating diet in relation to bone health has primarily focused on individual dietary nutrients, in particular calcium and vitamin D, and have formed the basis of many dietary guidelines which aim to prevent osteoporosis and fracture. Despite the fact that analysing individual nutrients has been common practice in previous research, the highly correlated nature of dietary exposures means it is difficult to determine the effect of individual nutrients and foods [1]. A dietary patterns approach considers the diet as a whole and has the ability to account for the potential interactive effects between nutrients and food groups turning the co-linearity of foods into an analytical advantage [2].

Previous research describing the relationship between dietary patterns and bone health in older adults have generally described a positive relationship between diets considered to be of higher overall quality and bone outcomes. In one of the earlier studies exploring dietary patterns and bone mineral density (BMD), a positive association was described in men between BMD and a dietary pattern characterised by high consumption of fruit and vegetables [3]. In the same study, poor diet, categorised as high consumption of sugar and confectionary, was negatively associated with BMD in both men and women [3]. Similarly, patterns describing high intakes of processed and snack foods were found to be associated with lower BMD at the femoral neck in postmenopausal Scottish women [4]. Research from the UK based National Survey of Health and Development described a positive association with a healthy dietary pattern rich in potassium, calcium and protein throughout adulthood and size-adjusted bone mineral content (BMC) and volumetric BMD at ages 60–64 years in women [5].

Dietary patterns have also been shown to be associated with fracture risk. Diets high in vegetables, fruit and whole grains were associated with reduced risk of low-trauma fracture in a population of Canadian men and women [6]. Two recent longitudinal studies have shown inverse associations with the Mediterranean Diet Score and the reduced risk of hip fractures in postmenopausal women from the United States [7] and Swedish men and women [8]. A similar dietary pattern based on intakes of fruit, vegetables and dairy foods was positively associated with BMD at baseline, as well as, lower risk of fractures at follow-up (median follow-up 14.8 years) [9]. Most recently, data from a large female Swedish cohort, with an average of 25.5 years of follow-up, reported a 31% reduced risk for women in the highest quartile of a healthy dietary pattern than those in the lowest quartile [10]. Associations between fracture risk and diet quality may also be due to diet being important for muscle strength and physical performance which would be preventative of falls and thus fractures. Improved muscle strength may also directly benefit bone strength through the relationship between muscle loading and bone outcomes. Several studies, including work in the cohort being presented here, show positive associations between muscle strength and pQCT outcomes [11, 12].

The majority of the studies investigating dietary patterns and bone health have mainly utilised dual-energy X-ray absorptiometry (DXA) [4–6, 9, 13] to measure BMC and BMD as surrogates for fracture risk. Advances in bone imaging techniques provide more detailed characterisation of bone. High-resolution peripheral quantitative computed tomography (HRpQCT) differentiates between cortical and trabecular compartments at distal sites and uniquely provide non-invasive estimates of bone microarchitecture. In addition, research has shown that HRpQCT has the potential to identify age-related changes and gender differences in bone microarchitecture [14, 15].

To the knowledge of the authors, no previous studies have explored the relationship between dietary patterns and measures of bone using HRpQCT.

The aim of this study was to assess the relationship between diet quality and BMD, bone geometry and microarchitecture measured by HRpQCT at the radius and tibia metaphysis and peripheral quantitative computed tomography (pQCT) at metaphyseal and diaphyseal sites, in community-dwelling older adults from the Hertfordshire Cohort.

## Methodology

The Hertfordshire Cohort Study (HCS) is a population-based cohort of older adults, consisting of 1579 men and 1418 women, born in Hertfordshire, UK, between 1931 and 1939 and still living in the county in 1998–2003. For each of these participants, we hold a set of detailed birth records which were maintained by Hertfordshire midwifes and hold the birth weight for each participant. Following our initial contact in 1998–2003, participants completed a baseline home interview and attended a research clinic for detailed assessment of their socio-demographic, lifestyle and clinical characteristics; the study has previously been described in detail [16].

Dietary data were collected at the home interview via a nurse-administered 129-item food frequency questionnaires (FFQ) at baseline. A ‘prudent’ diet score was calculated using principal component analysis [17]. The ‘prudent’ diet scores were standardised, with a mean of 0 and a standard deviation of 1, with a higher score reflecting an overall better quality of diet and a negative score representing a lower quality of diet. Higher-quality diets describe higher consumption of fruit, vegetables, whole grain cereals and oily fish and low consumption of white bread, added sugar, full-fat dairy products, chips and processed meat [17]. Further details about the process used to calculate the diet quality score have previously been published [17]. Energy intakes from the baseline FFQ were calculated by multiplying the frequency of consumption of a portion of each food by its nutrient content according to the UK national food composition database or manufacturers’ composition data. Smoking status, weekly consumption of alcohol and level of physical activity (Dallosso questionnaire) [18] were also ascertained by the nurse-administered questionnaire.

A subset of HCS baseline participants, residing in East Hertfordshire (*n* = 570), were invited to take part in a follow-up study in 2011–2012 which included a pQCT and HRpQCT scan of the non-dominant radius and tibia. PQCT was performed using a Stratec XCT2000 instrument, scans were acquired at the 4 and 66% sites of the radius, and at the 4 and 38% sites of the tibia. HRpQCT measures were taken using a Scanco XtremeCT scanner and evaluated using standard evaluation and cortical porosity scripts. Of those invited, 376 men and women agreed to participate. Complete dietary and HRpQCT data were available for 184 men and 166 women for these analyses. Due to the variations in the mean dietary quality scores, and previous evidence suggesting gender differences in bone microarchitecture [15], men and women were analysed separately.

Linear regression analysis was used to test for sex-specific associations in pQCT and HRpQCT outcomes. Models were conducted with and without pre-defined adjustments. Fully adjusted models included baseline measures of age, smoking status (never, ex-smoker or current smoker), alcohol consumption (in relation to gender-specific UK alcohol guidelines at the time of the data collection), physical activity level and height. Additional adjustments of years since menopause and HRT use at time of scan were made for women.

All statistical analyses were conducted using STATA 14.

## Results

### Participant Characteristics

Characteristics of the 350 HCS participants who were included in the analysis sample are presented in Table 1. Mean (SD) age at HCS baseline was 63.8 (2.5) and 65.5 (2.6) years among men and women, respectively. On average, women had higher dietary quality scores compared with men (Mean (SD) 0.62 (1.14) and − 0.24 (1.23), respectively). A higher proportion of men at baseline were either current or ex-smokers compared to women. Just over a quarter of men reported consuming more than the recommended levels of alcohol, whereas only 1.8% of females consumed more than the recommended number of weekly alcohol units. 42.6% of men and 44% of women were from a non-manual social class.

**Table 1:** Study participants characteristics

	Males (*n* = 184)		Females (*n* = 166)	
	Mean	SD	Mean	SD
Age (years)^a^	63.8	2.5	65.5	2.6
Age (years) at HRpQCT scan	76.1	2.5	76.4	2.6
Height (cm) at HRpQCT scan	173.5	6.4	159.9	5.7
BMI (kg/m^2^) at HRpQCT scan	27.5	3.7	28.0	4.7
HCS prudent diet score (based on 24 food items)^a^	− 0.24	1.23	0.62	1.1
Average daily energy intake (kcal)^a^	2382.6	494.1	1990.7	423.7
Dallosso physical activity score^a^	65.5	13.5	62.2	13.7

	n	%	n	%
Smoking status^a^				
Never	73	39.7	109	65.7
Ex	91	49.5	46	27.7
Current	20	10.9	11	6.6
Alcohol consumption^a^				
None	2	1.1	18	10.8
≤ recommended^b^	135	73.4	145	87.4
> recommended^b^	47	25.5	3	1.8
Social class^a^				
Non-manual	75	42.6	73	44
Manual	101	57.4	93	56
HRT use^a^				
No			90	54.2
Yes			76	45.8

^a^Measure taken at baseline

^b^Recommended maximum weekly consumption of alcohol (14 units for women, 21 units for men)

### Associations Between Diet Quality and Bone Health Measured Using HRpQCT

**Fig. 1:**
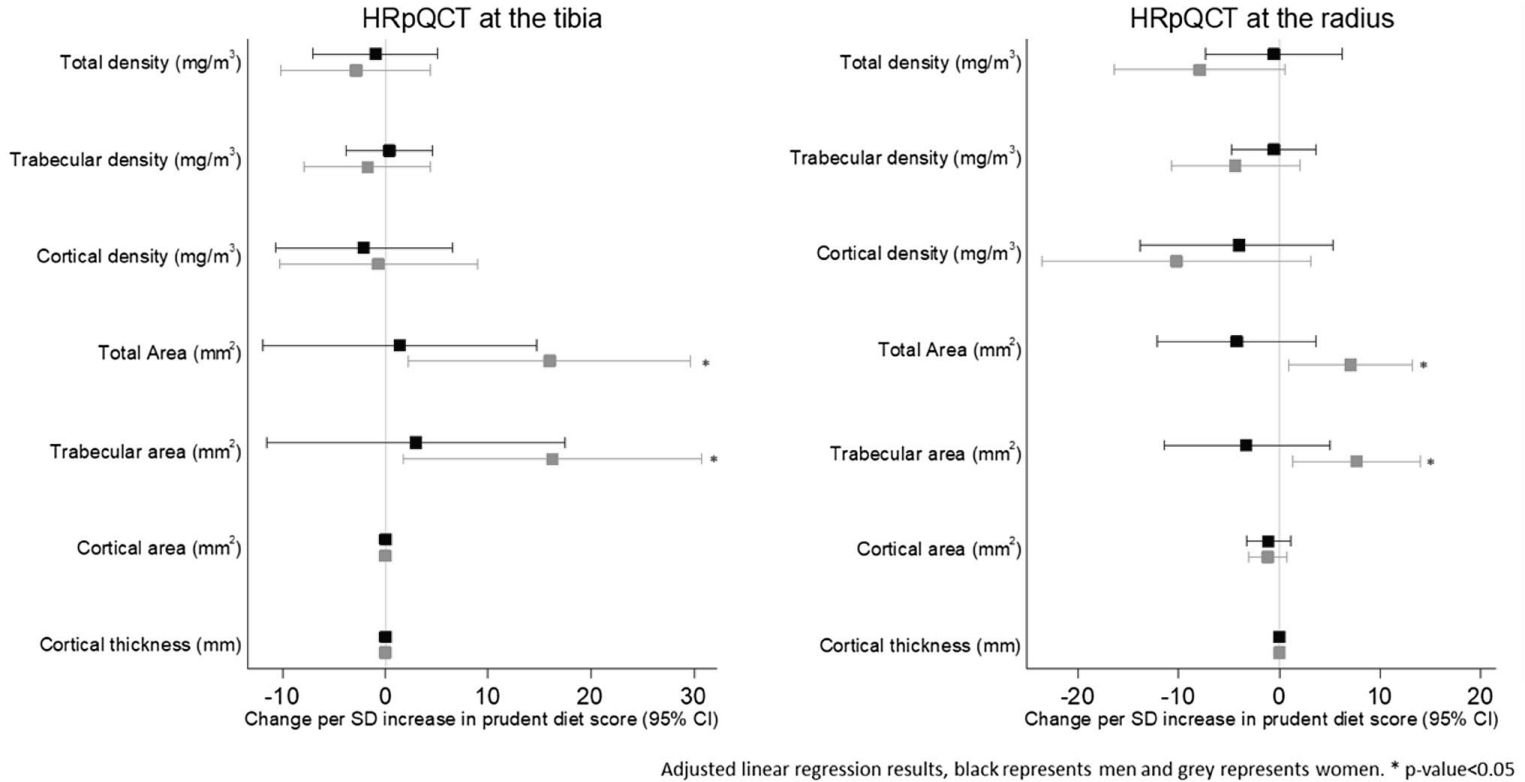
Linear regression results assessing the relationship between prudent diet and HRpQCT bone parameters

Associations between baseline dietary quality score and bone outcome measures using HRpQCT are presented in Fig. 1 (Supplementary Table 1). In women, a significant positive univariate relationship was found between baseline prudent diet score and total and trabecular area at both the radius and the tibia. These associations remained robust to adjustment. No significant associations were observed between baseline prudent diet score and cortical area, cortical thickness, cortical density or trabecular density at either of the measured sites in women. Although patterns were similar, no significant associations were observed in men.

### Associations Between Diet Quality and Bone Health Measured Using pQCT

**Fig. 2:**
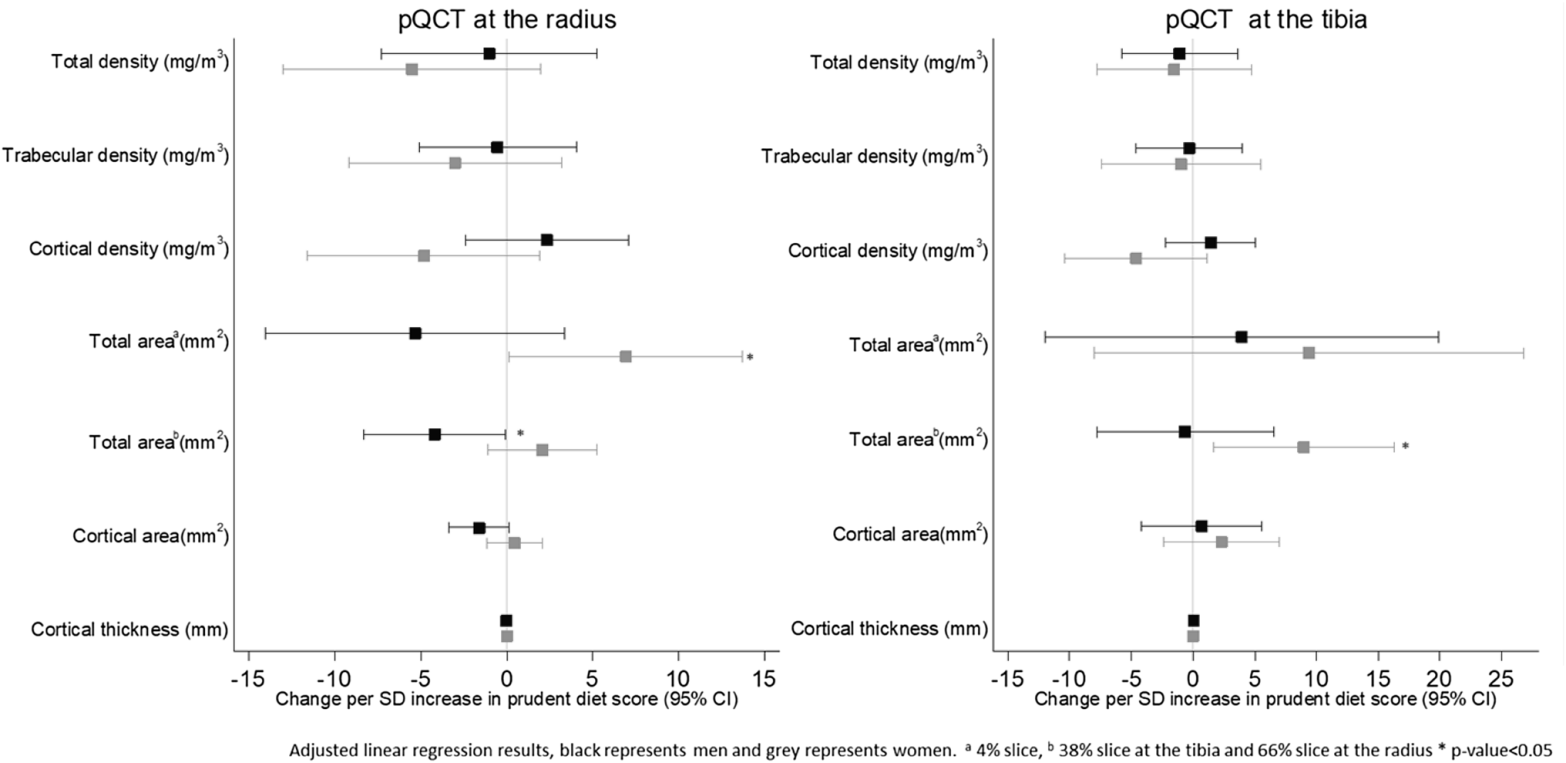
Linear regression results assessing the relationship between prudent diet and pQCT bone parameters

The associations between baseline dietary quality scores and bone outcomes as measured by pQCT for both men and women are presented in Fig. 2 (Supplementary Table 2). The results using bone parameters measured using pQCT show similar trends to the analysis using HRpQCT parameters, the 4% site being similar to the HRpQCT scan site. In women, significant positive associations were found for total area at the tibia (4% slice), the association remained after adjustments. At the tibia diaphysis (cortical site), significant positive associations were also found for total area and cortical area. In the fully adjusted models, the significant positive association between baseline dietary quality score and total area (38% slice) remained but all others were attenuated. At the radius, significant positive associations were found for total area (4% slice) and remained statistically significant after adjustment. Polar strength strain index (33% slice) was found to have a significant positive association with baseline diet quality score which remained after adjustment (*β* = 6.66, (95% CI 0.07–13.26), *p* = 0.05). For men, significant associations were observed at the radius for polar strength strain index (66% slice) (*β* = − 11.89, (95% CI − 22.33 to − 1.46), *p* = 0.03) and total area (66% slice) only after adjustment.

To exclude the possibility that body mass index (BMI) was the determinate for the observed differences in bone size, additional analyses were completed adjusting for BMI; the relationships between dietary patterns and bone parameters remained unchanged.

## Discussion

### Summary of Results

Using data from the HCS, we have shown that a higher dietary quality, representing diets consisting of greater intakes of fruit, vegetables, oily fish and whole grain cereals, and low consumption of white bread, added sugar, full-fat dairy products, chips and processed meat, is associated with greater bone size in women but not in men. Results using HRpQCT imaging showed significant associations in women for total and trabecular area at the metaphysis of both the radius and tibia. Associations between pQCT results and dietary pattern scores showed a similar pattern of results, at both the metaphysis and diaphysis, with a positive association being observed for the measures of cross-sectional area. Together the data suggest that diet quality in early old age predicted bone size at follow-up (median follow-up of 11.6 years) in women; no consistent relationships were found in men.

### Comparison to Previous Research

Previous research, utilising a dietary patterns approach to investigate associations with bone health, has found favourable associations between high-quality diets, mainly based on fruit and vegetables, and BMC and BMD [3–5, 13], using DXA to obtain measures of bone health. This is in contrast to the current findings which did not show any associations between volumetric trabecular, total and cortical bone density measured by HRpQCT and pQCT. To test whether the lack of agreement with previous studies was due to methodology used, the current analysis was repeated with DXA-derived areal BMD of the total hip and whole body. Relationships remained unchanged suggesting that in this cohort of older individuals diet quality seems to be affecting bone size rather than density.

The significant findings in women, but not in men, in this study may be due to the difference between the sexes in the levels of adherence to the dietary quality score between assessment and bone measurements in this cohort. The median time between dietary assessment and bone assessment was 11.6 years (range 9.9–13.6 years). These findings are similar to those in a study of the National Survey for Health and Development cohort, which also described a positive association between a protein–calcium–potassium rich dietary pattern and bone outcomes, though this time with BMD in women but not in men [5]. The protein–calcium–potassium rich pattern was characterised by high intakes of whole grains, fruits and vegetables, fish and low fat dairy and similar to the prudent diet score used in our study. The authors suggested a possible explanation to be that men were at a different stage of skeletal ageing than in women [5]. The HCS cohort was older than NSHD at the time of bone assessment. Despite this, the sex differences were consistent. Results may be indicative of females having greater awareness of diet and importance for health post-menopause.

Some studies report greater bone area being associated with higher fracture risk. Previous research using data from the HCS showed that, in men, there was a reduction in the odds of prevalent fracture for every one SD reduction in total area and trabecular area (Odds Ratio (OR) (95% CI) 0.50 (0.31–0.78) and 0.52 (0.34–0.78), respectively, *p* < 0.01 for both). In contrast, these relationships were not found in women from HCS, nor in the UK-arm of the GLOW cohort [12, 19]. It would be counterintuitive to suggest that a better diet quality has a negative impact on fracture risk. Instead, we suggest that the larger bones and better quality are indicative of better diets throughout life, particularly during growth, which would increase height and consequently bone area. Alternatively, previous research has described a positive association with age and total bone area that is more pronounced at certain bone sites in men than in women after adjustment for body size [20]. MacDonald and colleagues state that this increase in total bone area suggests continued periosteal apposition [20]. Our results may suggest that a better diet quality is associated with increased rates of periosteal apposition during adulthood in women and in turn, may result in greater bone area in later life which may preserve strength, and hence explain the lack of association between bone area by HRpQCT and fracture in women. Further research is required to understand these relationships; however, this should be explored through data acquired at multiple time points.

### Strengths and Limitations

Our study has a number of strengths. HRpQCT provides measurements of bone microstructure and has not been previously used to explore the relationship between bone and whole diet quality. Our study also has a number of limitations. The relatively small sample of participants who had HRpQCT, pQCT and dietary data limits the conclusions that can be made from this study.

A further limitation of this study is the fact that diet quality is only measured at one time point. Future studies would benefit from the use of multiple measures of dietary quality to explore if change in diet quality over time is related to a change in bone health. All participants were older Caucasian men and women from the UK which may limit any generalisability to wider populations; however, the HCS cohort has been shown to be broadly comparable with the study participants in the nationally representative Health Survey for England [16]. In addition, a ‘healthy’ responder bias is evident within the HCS [21]; however, it is unlikely to have affected the observed associations between dietary quality and bone parameters.

## Conclusions

To our knowledge, this is the first study to combine dietary pattern data with novel measures of bone parameters using both pQCT and HRpQCT data. Our data suggest that diets high in fruit, vegetables, oily fish and whole grain cereals in early old age are associated with greater bone size but not volumetric bone density or microarchitecture in later life in women. Further research is required with a larger sample to explore causality and to assess potential for dietary interventions in later life.

## Electronic supplementary material

Below is the link to the electronic supplementary material.

Supplementary material 1 (DOCX 23 KB)
