# Supplementary material for: Diet Quality and Bone Measurements Using HRpQCT and pQCT in Older Community-Dwelling Adults from the Hertfordshire Cohort Study
Source: Calcif Tissue Int. 2018 Jun 21;103(5):494–500. doi: 10.1007/s00223-018-0445-x (PMC6174074; doi:10.1007/s00223-018-0445-x)
Supplement: Supplementary file 1 — Supplementary material 1 (DOCX 23 KB) [file 223_2018_445_MOESM1_ESM.docx]

**Supplementary Table 1: Linear regression analysis assessing the relationship between prudent diet and bone parameters, from HRpQCT, in men and women in both the tibia and radius**

|  | **Men** | | | | **Women** | | | |
| --- | --- | --- | --- | --- | --- | --- | --- | --- |
|  | **Radius** | | **Tibia** | | **Radius** | | **Tibia** | |
|  | **Unadjusted** | **Adjusted ^a^** | **Unadjusted** | **Adjusted ^a^** | **Unadjusted** | **Adjusted ^b^** | **Unadjusted** | **Adjusted ^b^** |
|  | **Beta** | **Beta** | **Beta** | **Beta** | **Beta** | **Beta** | **Beta** | **Beta** |
|  | **95% C.I** | **95% C.I** | **95% C.I** | **95% C.I** | **95% C.I** | **95% C.I** | **95% C.I** | **95% C.I** |
|  | **p-value** | **p-value** | **p-value** | **p-value** | **p-value** | **p-value** | **p-value** | **p-value** |
| **Total density (mg/cm3)** | -1.19 | -0.61 | -2.49 | -0.98 | -5.33 | **-9.10** | -0.89 | -2.73 |
|  | (-7.91,5.52) | (-7.39,6.18) | (-8.60,3.62) | (-7.06,5.10) | (-13.56,2.90) | **(-17.98,-0.21)** | (-7.83,6.06) | (-10.16,4.69) |
|  | 0.73 | 0.86 | 0.42 | 0.75 | 0.20 | **0.04** | 0.80 | 0.47 |
|  |  |  |  |  |  |  |  |  |
| **Trabecular density (mg/cm3)** | -0.29 | -0.57 | 0.50 | 0.37 | -0.70 | -3.02 | 0.94 | -0.90 |
|  | (-4.48,3.90) | (-4.74,3.59) | (-3.62,4.61) | (-3.87,4.62) | (-6.66,5.26) | (-9.50,3.46) | (-4.64,6.53) | (-7.08,5.27) |
|  | 0.89 | 0.79 | 0.81 | 0.86 | 0.82 | 0.36 | 0.74 | 0.77 |
|  |  |  |  |  |  |  |  |  |
| **Cortical density (mg/cm^3^)** | -5.67 | -4.03 | -4.11 | -2.08 | -5.23 | -10.25 | 1.83 | -0.69 |
|  | (-14.69,3.36) | (-13.38,5.31) | (-12.49,4.27) | (-10.69,6.54) | (-17.12,6.67) | (-23.57,3.07) | (-7.15,10.82) | (-10.32,8.95) |
|  | 0.22 | 0.40 | 0.33 | 0.63 | 0.39 | 0.13 | 0.69 | 0.89 |
|  |  |  |  |  |  |  |  |  |
| **Total area (mm^2^)** | 0.18 | -4.26 | 11.41 | 1.40 | **8.34** | **7.02** | **22.69** | **15.97** |
|  | (-8.20,8.56) | (-12.12,3.60) | (-5.17,27.98) | (-11.93,14.72) | **(2.34,14.34)** | **(0.89,13.14)** | **(8.65,36.74)** | **(2.27,29.67)** |
|  | 0.97 | 0.29 | 0.18 | 0.84 | **0.01** | **0.03** | **0.00** | **0.02** |
|  |  |  |  |  |  |  |  |  |
| **Trabecular area (mm^2^)** | 0.95 | -3.31 | 13.36 | 2.98 | **8.37** | **7.64** | **21.98** | **16.23** |
|  | (-7.68,9.58) | (-11.53,4.92) | (-4.24,30.97) | (-11.50,17.47) | **(2.22,14.53)** | **(1.30,13.98)** | **(6.99,36.97)** | **(1.68,30.78)** |
|  | 0.83 | 0.43 | 0.14 | 0.68 | **0.01** | **0.02** | **0.00** | **0.03** |
|  |  |  |  |  |  |  |  |  |
| **Cortical area (mm^2^)** | -1.07 | -1.09 | -2.08 | -1.57 | -0.35 | -1.20 | 0.98 | -0.19 |
|  | (-3.20,1.06) | (-3.26,1.09) | (-5.98,1.81) | (-5.59,2.46) | (-2.03,1.33) | (-3.09,0.69) | (-2.18,4.14) | (-3.57,3.19) |
|  | 0.32 | 0.33 | 0.29 | 0.44 | 0.68 | 0.21 | 0.54 | 0.91 |
|  |  |  |  |  |  |  |  |  |
| **Cortical thickness (mm)** | -0.01 | -0.01 | -0.03 | -0.02 | -0.01 | -0.03 | 0.00 | -0.01 |
|  | (-0.04,0.01) | (-0.04,0.01) | (-0.06,0.01) | (-0.05,0.02) | (-0.04,0.01) | (-0.05,0.00) | (-0.04,0.03) | (-0.04,0.03) |
|  | 0.26 | 0.38 | 0.15 | 0.39 | 0.24 | 0.07 | 0.91 | 0.60 |

^a^ Adjusted for age at scan, smoking status, alcohol consumption, height at scan and physical activity; ^b^ Adjusted for age at scan, smoking status, alcohol consumption, height at scan, physical activity, HRT use and years since menopause

**Supplementary Table 2: Linear regression analysis assessing the relationship between prudent diet and bone parameters, from pQCT, in men and women in both the tibia and radius**

|  | **Men** | | | | **Women** | | | |
| --- | --- | --- | --- | --- | --- | --- | --- | --- |
|  | **Radius** | | **Tibia** | | **Radius** | | **Tibia** | |
|  | **Unadjusted** | **Adjusted ^a^** | **Unadjusted** | **Adjusted ^a^** | **Unadjusted** | **Adjusted ^b^** | **Unadjusted** | **Adjusted ^b^** |
|  | **Beta** | **Beta** | **Beta** | **Beta** | **Beta** | **Beta** | **Beta** | **Beta** |
|  | **95% C.I** | **95% C.I** | **95% C.I** | **95% C.I** | **95% C.I** | **95% C.I** | **95% C.I** | **95% C.I** |
|  | **p-value** | **p-value** | **p-value** | **p-value** | **p-value** | **p-value** | **p-value** | **p-value** |
| **Total density 4% (mg/cm3)** | -0.54 | -1.01 | -1.73 | -1.09 | -1.72 | -5.49 | 1.23 | -1.56 |
|  | (-6.76,5.68) | (-7.32,5.29) | (-6.39,2.93) | (-5.81,3.63) | (-8.53,5.10) | (-12.97,1.98) | (-4.59,7.04) | (-7.80,4.68) |
|  | 0.86 | 0.75 | 0.46 | 0.65 | 0.62 | 0.15 | 0.68 | 0.62 |
|  |  |  |  |  |  |  |  |  |
| **Trabecular density 4% (mg/cm3)** | -0.58 | -0.52 | -0.67 | -0.32 | -1.00 | -3.00 | 1.23 | -0.97 |
|  | (-5.17,4.00) | (-5.11,4.06) | (-4.88,3.53) | (-4.63,3.99) | (-6.74,4.74) | (-9.18,3.19) | (-4.73,7.19) | (-7.39,5.45) |
|  | 0.80 | 0.82 | 0.75 | 0.88 | 0.73 | 0.34 | 0.68 | 0.77 |
|  |  |  |  |  |  |  |  |  |
| **Cortical density ^c^ (mg/cm^3^)** | 2.30 | 2.33 | 1.78 | 1.43 | -3.16 | -4.82 | -4.93 | -4.64 |
|  | (-2.52,7.11) | (-2.40,7.07) | (-1.76,5.33) | (-2.21,5.08) | (-9.22,2.91) | (-11.58,1.94) | (-10.03,0.18) | (-10.42,1.13) |
|  | 0.35 | 0.33 | 0.32 | 0.44 | 0.31 | 0.16 | 0.06 | 0.11 |
|  |  |  |  |  |  |  |  |  |
| **Total area 4% (mm^2^)** | -2.69 | -5.32 | 12.14 | 3.94 | **6.78** | **6.93** | **16.93** | 9.37 |
|  | (-11.46,6.07) | (-14.01,3.38) | (-5.94,30.22) | (-12.02,19.90) | **(0.43,13.12)** | **(0.12,13.73)** | **(0.45,33.42)** | (-8.03,26.77) |
|  | 0.55 | 0.23 | 0.19 | 0.63 | **0.04** | **0.05** | **0.04** | 0.29 |
|  |  |  |  |  |  |  |  |  |
| **Total area ^c^ (mm^2^)** | -3.35 | **-4.20** | 1.28 | -0.67 | 2.33 | 2.06 | **11.53** | **8.95** |
|  | (-7.53,0.84) | **(-8.31,-0.09)** | (-6.36,8.91) | (-7.82,6.47) | (-0.52,5.18) | (-1.11,5.24) | **(4.72,18.34)** | **(1.63,16.28)** |
|  | 0.12 | **0.05** | 0.74 | 0.85 | 0.11 | 0.20 | **<0.01** | **0.02** |
|  |  |  |  |  |  |  |  |  |
| **Cortical area ^c^ (mm^2^)** | -1.14 | -1.61 | 1.90 | 0.68 | 1.03 | 0.47 | **4.88** | 2.29 |
|  | (-2.95,0.66) | (-3.39,0.16) | (-3.03,6.82) | (-4.17,5.54) | (-0.46,2.53) | (-1.15,2.09) | **(0.56,9.20)** | (-2.40,6.99) |
|  | 0.21 | 0.07 | 0.45 | 0.78 | 0.17 | 0.57 | **0.03** | 0.34 |
|  |  |  |  |  |  |  |  |  |
| **Cortical thickness ^c^ (mm)** | -0.01 | -0.01 | 0.02 | 0.01 | 0.01 | 0.00 | 0.02 | -0.01 |
|  | (-0.05,0.04) | (-0.05,0.03) | (-0.05,0.10) | (-0.06,0.09) | (-0.03,0.06) | (-0.05,0.05) | (-0.06,0.10) | (-0.11,0.08) |
|  | 0.78 | 0.59 | 0.56 | 0.77 | 0.54 | 0.98 | 0.67 | 0.75 |
|  |  |  |  |  |  |  |  |  |
| **Polar strength strain index ^c^ (mm^3^)** | -8.70 | **-11.89** | 19.27 | 8.16 | **7.99** | **6.66** | **47.02** | 29.91 |
|  | (-19.11,1.70) | **(-22.33,-1.46)** | (-17.94,56.48) | (-27.10,43.41) | **(1.82,14.16)** | **(0.07,13.26)** | **(18.63,75.42)** | (-0.69,60.50) |
|  | 0.10 | **0.03** | 0.31 | 0.65 | **0.01** | **0.05** | **<0.01** | 0.06 |

^a^ Adjusted for age at scan, smoking status, alcohol consumption, height at scan and physical activity; ^b^ Adjusted for age at scan, smoking status, alcohol consumption, height at scan, physical activity, HRT use and years since menopause; ^c^ 38% slice in the tibia and 66% slice in the radius
